# Application of crop wild relatives in modern breeding: An overview of resources, experimental and computational methodologies

**DOI:** 10.3389/fpls.2022.1008904

**Published:** 2022-11-17

**Authors:** Soodeh Tirnaz, Jaco Zandberg, William J. W. Thomas, Jacob Marsh, David Edwards, Jacqueline Batley

**Affiliations:** School of Biological Sciences, University of Western Australia, Perth, WA, Australia

**Keywords:** pangenome, wild species, modern breeding, ex situ resources, *in situ* resources

## Abstract

Global agricultural industries are under pressure to meet the future food demand; however, the existing crop genetic diversity might not be sufficient to meet this expectation. Advances in genome sequencing technologies and availability of reference genomes for over 300 plant species reveals the hidden genetic diversity in crop wild relatives (CWRs), which could have significant impacts in crop improvement. There are many *ex-situ* and *in-situ* resources around the world holding rare and valuable wild species, of which many carry agronomically important traits and it is crucial for users to be aware of their availability. Here we aim to explore the available *ex-/in- situ* resources such as genebanks, botanical gardens, national parks, conservation hotspots and inventories holding CWR accessions. In addition we highlight the advances in availability and use of CWR genomic resources, such as their contribution in pangenome construction and introducing novel genes into crops. We also discuss the potential and challenges of modern breeding experimental approaches (e.g. *de novo* domestication, genome editing and speed breeding) used in CWRs and the use of computational (e.g. machine learning) approaches that could speed up utilization of CWR species in breeding programs towards crop adaptability and yield improvement.

## What can CWRs offer?

The world population is estimated to come close to 10 billion by 2050, while a food gap of more 50% is expected between 2006 and 2050 (Ranganathan et al., 2016). In addition, the growing consequences of climate change, such as increasing weed prevalence and the occurrence of severe disease epidemics and drought stresses (Raza et al., 2019) will lead towards billions of dollars of crop yield losses worldwide (Gregory et al., 2009; Mittler and Blumwald, 2010). The IPCC (2014) has projected yield losses of up to 25% due to climate change if crop adaptation and improvement are not implemented (IPCC, 2014). At the same time, diets are changing, with shifting nutritional demands toward gluten free, plant-based protein and low GI (glycaemic index) products (Gaikwad et al., 2020). As a result, there is an urgent need for plant breeders to develop new traits in addition to agronomically important traits such as disease resistance, drought tolerance, and yield improvements. On top of these challenges, the effect of the recent COVID-19 pandemic on future agricultural industries has likely added financial strain to both production and distribution chains due to restricted food trade policies and closure of food production facilities (Aday and Aday, 2020). These factors put farmers in a precarious position, with growing pressure to increase production, while they are placed in an increasingly vulnerable position to crop failure and infrastructure setbacks.

Providing breeders access to diverse genetic resources is essential to facilitate, accelerate and optimise crop improvement approaches while domestication bottlenecks have also restricted modern breeding populations (Allaby et al., 2019). The reduction in genetic diversity induced by domestication bottleneck is well documented among many crops such as common bean (Gepts et al., 1986; Papa and Gepts, 2003). Compared to the domesticated population, there are tremendous genetic diversity persists among crop wild relatives (CWRs). The structure of genetic diversity among wild populations appears to be stronger than domesticated; for example in common bean, the diversity of domesticated beans showed limited geographical structure and much less differentiation among populations and regions while in wild bean population even geographically-short-distanced populations carry significant genetic diversity (Papa and Gepts, 2003). As a result, the addition of CWRs to the current breeding programs can significantly widen the source of genetic variation and selection towards yield, resistance and nutritional quality improvement in crops. CWRs can be defined as any taxon belonging to the same genus as a crop; however this definition will include species that are both closely or remotely related to crops (Maxted et al., 2006). In a narrower definition CWRs belong to the same genus of the crop and are closely related to the crops (i.e they are ranked as same the species or same subgenus) (Maxted et al., 2006; Perrino and Perrino, 2020). Advances in breeding techniques, such as genome sequencing, pangenome construction and *de novo* domestication, have been facilitating traits/gene selection from both closely and remotely, related species where fertility and compatibility will be a barrier in traditional breeding approaches, related CWRs to crops. There are a number of successful examples of CWRs application in breeding, such as disease and pest resistance improvement in wheat, rice, potato, tomato, cassava, sunflower, banana and lettuce; yield improvement in wheat and rice; and improving tolerance to abiotic stress in rice, tomato, barley and chickpea (Hajjar and Hodgkin, 2007). CWRs have also contributed beneficial traits related to ideal plant architecture and weed suppression in rice (Inagaki et al., 2021).

The diversity among CWRs could also be used to decrease the rate of gene/genetic erosion, which has been happening over decades of crop domestication and intense breeding (Schouten et al., 2019). The FAO estimates that ~75% of the genetic diversity in crop varieties has been lost over the past century (FAO, 1999; Khoury et al., 2022). Genetic erosion restricts breeders by limiting sources of selection for identifying desirable agronomic traits. For instance, 96% of peas grown in the US originated from only 9 varieties (Esquinas-Alcázar, 2005). This limited genetic pool will significantly decrease diversity for natural and artificial selection, and intensify the vulnerability of modified varieties to rapid climate changes and new environmental stresses (Esquinas-Alcázar, 2005). Pangenomic analyses in soybean also revealed a reduction in mean gene count per individual due to domestication (Bayer et al., 2022), with disproportionately high levels of biotic and abiotic stress genes lost in modern breeding populations compared to CWRs (Liu et al., 2020). Fortunately, the application of wild species in breeding programs can be used to recover lost diversity caused by erosion, and boost diversity among the crops. SNP array analysis showed that genetic diversity among commercial tomato varieties (from NW Europe) increased by a factor of eight over 7 decades (starting from the 1950s) as a result of the introgression of many disease resistances genes from wild relatives (Schouten et al., 2019).

The application of CWRs in breeding has been also shown to deliver huge economic returns in agricultural industries worldwide, with their annual contribution to the world economy estimated at around US $186.3 billion in 2020 (Tyack et al., 2020; Bohra et al., 2022). It has been estimated that around 30% of crop yield improvement since 1945, valued worldwide at around US $100 billion, is a result of CWR use in crop breeding (Pimentel et al., 1997; Brozynska et al., 2016). In tomato, one wild variety provided genes increasing solids content by 2.4% which was worth US$250 million a year to the global tomato industry; and genes from three wild peanut varieties increased resistance to the root knot nematode, for potential savings of around US $100 million each year worldwide (Maxted, 2008).

Despite all the potential that CWRs can offer to improve breeding programs, their *in-situ* (in their natural habitats) and *ex-situ* (outside their natural habitats) conservation has been neglected over many years, leading to their potential extinction. Global and local studies have been conducted to guide CWR conservation strategies and estimate the potential loss of diversity of CWRs if the required actions have not been taken. In the US, conservation assessments for 600 CWRs show 42 taxa (7%) are critically endangered in their natural habitats, 297 (50%) are endangered, 166 (28%) are vulnerable, 66 (11%) are near threatened, and only 23 (3%) are of least concern (Khoury Colin et al., 2020). Another CWR conservation study revealed that the diversity of CWRs is poorly represented in genebanks while out of 1,076 taxa related to 81 crops, for 313 (29%) taxa no germplasm accessions exist, and for 257 (23%) taxa fewer than ten accessions exist (Castañeda-Álvarez et al., 2016). A conservation study on 29 threatened CWRs in Italy, also indicates 23 out of 29 species, have no gene pool at all. In addition, there is not enough data of their *ex-situ* and *in-situ* conservation while 16 and 22 species were identified as high priority for *ex-situ* and *in-situ* conservation respectively (Perrino and Wagensommer, 2022).

Rapid advancements in sequencing technology and computational approaches offer excellent opportunities to fully harness CWR diversity for crop improvement. However, the availability and accessibility of the existing CWR genebank and germplasm resources, capability of modern breeding methodologies and techniques in use of CWRs conservation strategies are currently not well developed to support their full potential and contribution in the current breeding programs. In this regard, here we discuss available in-/*ex-situ* resources for the preservation of CWR variation and the advances in the modern experimental methodologies and computational tools to facilitate capturing the genetic diversity among CWR and their utilization in breeding.

## Ex-situ resources

*Ex-situ* resources, e.g. genebanks and botanical gardens, facilitate user access to plant samples without the need for collecting samples directly from their natural habitat, which can be laborious and complicated when species only exist in remote locations and in most cases need collecting permit (PolicyReport, 2016) and in many cases may not accessible because of political or socio-economic unrest. The number of accessions held worldwide in genebanks estimated at ~7.4 million accessions in 2009, which increased more than 1.4 million from 1996, ~30% of this increase associated with CWR (van Bemmelen van der Plaat et al., 2021). There are now more than 1750 genebanks worldwide, with 130 of them holding more than 10,000 accessions each (Bohra et al., 2021). Wheat (856,168 accessions), rice (773,948 accessions), barley (466,531 accessions), maize (327,932 accessions) and bean (261,963 accessions) are the most represented crops across the world’s genebanks (Wambugu et al., 2018).

To facilitate global access and the conservation of genetic diversity of cultivated and CWR species, genebanks work collaboratively; for instance, Genesys is a database (platform) that contains information of around 4 million accessions across 450 institutes and allows researchers, breeders and policymakers to browse across all genebanks (https://www.genesys-pgr.org/content/about/about ) (**Table 1**). The Genesys database also includes accession information of three of the world’s largest genebank databases; the Consultative Group on International Agricultural Research (CGIAR), European Search Catalogue for Plant Genetic Resources (EURISCO), and the U.S. National Plant Germplasm System (NPGS). In contrast to CGIAR and EURISCO that hold both crops and CWRs accessions, the NPGS collection mainly focuses on crop germplasm (https://www.ars-grin.gov/Pages/Collections#bkmk-1 ). The EURISCO database contains over 2 million accessions of crop plants and their wild relatives preserved ex situ by about 400 institutes (https://eurisco.ipk-gatersleben.de/apex/eurisco_ws/r/eurisco/home ). CGIAR is a partnership of 11 genebanks conserving over 700,000 accessions of cereals, grain legumes, forages, tree species, root and tuber crops and banana and their wild relatives (**Table 1**). For instance, one of the CGIAR genebank partners is the International Institute of Tropical Agriculture (IITA) which holds over 28,000 accessions of plant material or germplasm of major African crops, including cassava, plantain and banana, yam, soybean, bambara ground-nut and maize. IITA holds the world’s largest collection of cowpeas, with 15,1222 samples from 88 countries, representing almost half of the global diversity (https://www.iita.org/research/genetic-resources/ ). There are also several genebanks that hold local genetic diversity of crop wild relatives, for example, the Karlsruher Institute of Technology (KIT) collected around 250 species of CWRs with 4500 accessions from all over Germany (https://www.botanik.kit.edu/garten/english/1056.php) (**Table 1**).

**Table 1:** A list of well-known resources and platforms that include CWR species.

Type	Platform/resource	Details	Reference/Access
Ex situ: Genebanks	KIT	4500 accessions of CWR across Germany	https://www.botanik.kit.edu/garten/english/1056.ph
	APG	70,000 accessions of pasture and forage	https://pir.sa.gov.au/research/australian_pastures_genebank
	World Vegetable Center	Holding 64,948 accessions which represent 330 species from 155 countries. Species are globally important vegetables such as tomato, onion, peppers, and cabbage as well as more than 10,000 accessions of traditional vegetables	https://avrdc.org/
	Genesys	Partnership with the 3 following genebanks	https://www.genesys-pgr.org/
	&Eurisco	Includes hundreds of research centers, genebanks and institutions across Europe	https://eurisco.ipk-gatersleben.de/apex/f?p=103:1
	&NPGS	Includes information of genebanks across U.S., managed by United States Department of Agriculture (USDA). NPGS comprises 20 separate institutions involved in plant germplasm collection, preservation, and distribution.	https://www.ars-grin.gov/Pages/Collections
	&CGIAR	Parentship with the following 11 genebanks	
	&African Rice Center	Holding almost 22,000 rice accessions, 85% of which originated in Africa	https://www.genebanks.org/genebanks/africarice/
	&Bioversity International	Holding the world’s largest collection of banana diversity, including more than 1,500 accessions of cultivated and wild species	https://www.genebanks.org/genebanks/biodiversity-international/
	&CIAT	Holding diverse collections of beans and tropical forages as seed and whole plants, and cassava *in vitro* and as small plants	https://www.genebanks.org/genebanks/ciat/
	&CIMMYT	Holding large collections of maize (28,000 accessions), including wild teosinte and Tripsacum wild relatives of maize and large collection of wheat (150,000 accessions) including landraces and wild relatives.	https://www.genebanks.org/genebanks/cimmyt/
	&CIP	The world’s largest collection of Potato and sweet potato and contains nearly all of the potato wild relatives	https://www.genebanks.org/genebanks/international-potato-centre/
	&ICARDA	Holding diverse collections of barley and wheat, grain legumes and forages, mostly traditional landraces and wild species from the Fertile Crescent	https://www.genebanks.org/genebanks/icarda/
	&ICRAF	Holding 190 species of wild, partially domesticated and domesticated trees	https://www.genebanks.org/genebanks/icraf/
	&ICRISAT	Holds more than 123,000 accessions of cultivated and wild relatives of pulses and cereals, including chickpea, sorghum and pigeonpea	https://www.genebanks.org/genebanks/icrisat/
	&ILRI	Collection of nearly 20,000 forage accessions, of which 97% are wild species	https://www.genebanks.org/genebanks/ilri/
	&IRRI	The largest collection of rice diversity in the world, with more than 130,000 accessions, including genetic stocks, landraces and wild relatives	https://www.genebanks.org/genebanks/irri/
	&IITA	Holding a collection of important African crops, including Bambara groundnut, cowpea, maize, soybean	https://www.genebanks.org/genebanks/iita/
Ex situ: Botanical gardens	BGCI	Including various databases such as PlantSearch, GardenSearch, ThreatSearch and GlobalTreeSearch	https://tools.bgci.org/garden_search.php
	BGCI-US	Botanic Gardens Conservation International and United States Botanic Garden collaboration effort identifying 22 global and 108 priority CWRs not reported in crop gene banks.	(Meyer and Barton., 2019)
*In situ*	ECPGR	Comprehensive concept for *in situ* conservation of CWR in Europe	https://www.ecpgr.cgiar.org/
	National Parks in India	Complete list of 103 national parks found in India currently under the protection of the Government	https://www.careerpower.in/national-parks-india.html
	RBGSYD	Australian *in situ* conservation site containing several key CWRs such as Macadamia nut, finger lime, etc	https://www.rbgsyd.nsw.gov.au/
	UNESCO	Biosphere reserves for the promotion of conserving biodiversity with sustainable use	https://en.unesco.org/biosphere
Software/tools	GRIN-Global	Genebank information management system (open-sourced software)	(Postman et al., 2009) https://www.grin-global.org/
	CWPs	CWR phylogenetic classification system	(Viruel et al., 2021)
	plaBiPD	Phylogenetic relations among flowering plants with published genome sequences	https://www.plabipd.de/plant_genomes_pa.ep
	Mercator	plaBiPD associated protein annotation tool	https://www.plabipd.de/mercator_about.html
	Nordic	Provides towards the planning and implementation of active *in-situ* and *ex-situ* conservation of CWR at a national level	http://www.cropwildrelatives.org/conservation-toolkit/introduction/
	GBIF	The Global Biodiversity Information Facility is a global database for the distribution of crop wild relatives with over 5 million records (34% germplasm records, 66% herbarium records)	https://www.gbif.org/dataset/07044577-bd82-4089-9f3a-f4a9d2170b2e
	ECP-GR Natura 2000	A tool for protected area managers of Europe to help identify which CWR genera are likely to occur in the protected areas	https://www.ecpgr.cgiar.org/crop-wild-relatives-in-natura-2000
Genomic databases	PLAZA – Monocots	Access to whole genome sequencing data	https://bioinformatics.psb.ugent.be/plaza/versions/plaza_v5_monocots/
	PLAZA - Dicots	Access to whole genome sequencing data	https://bioinformatics.psb.ugent.be/plaza/versions/plaza_v5_dicots/
	Germinate	A generic plant genetic database with several CWRs	https://germinateplatform.github.io/get-germinate/
	NCBI	General database for literature, genes, genomes and protein sequences of CWRs (Not-CWR specific)	https://www.ncbi.nlm.nih.gov /
	CerealsDB	SNP database for wheat	https://www.cerealsdb.uk.net/cerealgenomics/CerealsDB/indexNEW.php
	Brassica Information Portal (BIP)	Brassica specific	https://www.brassica.info/
	Genome Database for Rosaceae (GDR)	Rosaceae specific	https://www.rosaceae.org/
	GenRes Gateway	Access point to the European genetic resources for plants, forests and animals (including CWR)	https://www.genres.eu/
	ECPGR Central crop database	A database containing passport data, characteristics and primary evaluation data of the major collections of the respective crops.	https://www.ecpgr.cgiar.org/resources/germplasm- databases/ecpgr-central-crop-databases

International Center for Tropical Agriculture (CIAT), International Maize and Wheat Improvement Center (CIMMYT), International Potato Center (CIP), International Center for Agricultural Research in the Dry Areas (ICARDA), World Agroforestry (ICRAF), International Crops Research Institute for the Semi-Arid Tropics (ICRISAT), International Livestock Research Institute (ILRI), International Rice Research Institute (IRRI) and International Institute of Tropical Agriculture (IITA), European Search Catalogue for Plant Genetic Resources (EURISCO) and the National Plant Germplasm System (NPGS), Botanic Gardens Conservation International (BGCI), Crop wild phylorelatives (CWPs), The International Center for Agricultural Research in the Dry Areas (ICARDA), NCBI – National Center for Biotechnology information.

Recourses available in genebanks have been used in a number of studies, for example Abdallah et al. (2020) obtained 285 accessions, representing 13 *Lathyrus* (grass pea) species, from The International Center for Agricultural Research in the Dry Areas (ICARDA) and showed that wild *Lathyrus* species have higher resistance to broomrape weeds (*Orobanche* spp.), a root holoparasitic plant that causes significant damage to legume crops (Abdallah et al., 2021). Dida et al. (2021) obtained 52 finger millet accessions, including landraces, wild lines and hybrids between wild and cultivated genotypes, from the International Crops Research Institute for the Semi-Arid Tropics (ICRISAT) and Genetic Resources Research Institute (GeRRI) genebanks and found that wild accessions were more resistant to blast disease, caused by the *Magnaporthe grisea* fungus, in comparison to the cultivated accessions (Dida et al., 2021).

In addition to the germplasm conservation, there are also genebanks that provide seed kits to smallholder farmers to improve local access to the crop diversity towards better nutrition and supporting climate-resilient agriculture these also assist with the improvement of local genetic diversity among crops. For example, the World Vegetable Center (WorldVeg) genebank distributed over 42,000 seed kits, containing over 183,000 vegetable seeds, to smallholder farmers in Tanzania, Kenya and Uganda, between 2013 and 2017. The kits contained seed of promising accessions and open-pollinated breeding lines of traditional African vegetables, tomato, Capsicum pepper and soybean. The results show that introduced diversity through seed kits effectively improved local nutrition by facilitating access to various vegetables and also the introduction of new germplasm may slow down genetic erosion and enhance local vegetable diversity (Stoilova et al., 2019).

One of the main concerns across genebanks is the misclassification of species, as previously species identification was mostly based on morphological traits. However, recently the combination of traditional methods combined with molecular approaches, such as DNA barcoding, have improved the accuracy of species identification (van Bemmelen van der Plaat et al., 2021). For example, Mason et al., 2015 proved high-throughput genotyping approaches, such as a SNP array, is an effective methodology for species confirmation. They performed diversity assessment, using the Illumina Brassica 60K SNP array, across 180 Brassicaceae samples sourced from the Australian Grains Genebank and showed 76 of samples were misclassified (Mason et al., 2015). Through advances in genome sequencing technology and introduction of marker assisted breeding, the use of CWRs has intensified and with this growing interest it is important to keep information in the genebanks well documented and accurate. This is particularly important for use of CWRs in breeding programs where the success rate is highly dependent on the genetic distance between the species, particularly in approaches where crossing compatibility is important, it is crucial to have accurate information regarding the species taxonomy.

Botanical gardens are another *ex-situ* resource for germplasm; moreover they play a crucial role in preventing species extinction through integrated conservation actions (Mounce et al., 2017). Mounce et al. (2017) showed that botanic gardens contribute to the conservation of at least 105,634 species, representing 30% of all plant species diversity, including over 41% of known threatened species (Mounce et al., 2017). The Botanic Gardens Conservation International (BGCI) has the largest collection of living plants (**Table 1**). The GardenSearch database, within BGCI, is the only global source for botanical gardens and includes information on over 3,755 botanical institutions worldwide. GardenSearch allows users to search botanical gardens based on their location (country) and their specific features or expertise (https://tools.bgci.org/garden_search.php ). For example, based on information stored in GardenSearch, the botanical garden of South Australia has a collection of 40% of Australian flora including drought and salt tolerant plants. This information can facilitate the access and identification of plants with traits of interest for both breeding and research purposes. PlantSearch within the BGCI searches across 1,582,767 collection records, representing 642,718 taxa, at 1,194 institutions; in addition with Plant Search there is a specific option for CWR search at the taxa level (https://tools.bgci.org/plant_search.php ) (Mounce et al., 2017).

## In-situ resources

In contrast to *ex-situ* conservation sites, *in-situ* sites are typically natural habitats which are rarely curated, for example conservation/rehabilitation facilities or national parks. The benefit of *in-situ* resources is that they are genetically dynamic and continue to evolve in response to both natural and artificial selection, thereby enhancing their adaptation to the environments in which they are grown (Phillips et al., 2016). However, these *in-situ* collections are vulnerable to habitat destruction and/or encroachment caused by civil strife, human settlement pressure and natural disasters including wildfires, flooding, drought and volcanic eruptions. As such, the development of effective CWR conservation strategies is required nationally and globally. Several nations have already prioritised *in situ* CWR conservation, for example, Cyprus (178 priority CWR taxa) (Phillips et al., 2014), UK (148 priority CWR taxa) (Maxted et al., 2007; Fielder et al., 2015), US (821 priority CWR taxa) (Khoury et al., 2013; Khoury et al., 2019), Mexico (310 priority CWR taxa) (Contreras-Toledo et al, 2018), Czech (238 priority CWR taxa) (Taylor et al., 2013) and Norway (204 priority CWR taxa) (Phillips et al., 2016). These *in-situ* conservation efforts provide an ongoing roadmap for the study of the evolutionary history of the plant, which can provide insight into the persistence of traits, identification of new agriculturally significant traits and maintaining biodiversity (Khoury Colin et al., 2020). However, the incorporation of CWRs into traditional farming systems must be carefully considered as it may lead to unfavourable outcomes, for example, a study by Bernal et al., 2019., found that by incorporating a secluded maize genotype (Zea diploperennis) into Mexican and Argentinian farms, the pest ‘corn leafhopper’ was able to emerge as a widespread pest to corn farmers (Bernal et al., 2019).

Furthermore, CWR *in-situ* sites typically overlap with regions of high biodiversity, for example, as described by Vincent et al. (2022), the identified Mediterranean basin CWR hotspot shared 91% of its area with a region of high biodiversity, similarly, the California Floristic Province shared 90% between the CWR and biodiversity hotspots. This overlap has since been harnessed to aid in crop diversity and improvement studies, for example, the Unesco biosphere reserves promote solutions that reconcile the conservation of biodiversity with sustainable development (Benz et al., 2000). However, it is important to consider that *in-situ* resources should not only be limited to ‘wild’ regions. Traditional farming systems are not closed and isolated from gene flow, Louette et al., 1997., showed that the maize varieties cultivated by farmers of Cuzalapa, Mexico, changes in composition over time (Iltis et al., 1979; Louette et al., 1997). Despite certain changes to the germplasm being permanent, for example, the teosinte germplasm in maize which persists during advanced generations of backcrossing (Kato and Sanchez, 2002). In addition to the biodiversity hotspots, centers of origin/diversity, defined as global crop domestication regions including high diversity of both crops and their wild relatives (Vavilov, 1926), could be used as major sources for identification of CWRs. These diversity centers/regions include China; India; Indo-Malayan; Inner Asiatic; Mediterranean; Ethiopian; Central American; the Peruvian-Ecuadorian-Bolivian center, with sub-centers in both Chiloe, Chile and around the Brazil-Paraguay border (Vavilov et al., 1992; Pironon et al., 2020; Maxted and Vincent, 2021). Recently, by assessing the distribution of 222 major international crops and 2,731 of their wild relatives, including both closely and distant related wild species to the crops, Pironon et al. showed geographic distribution of major crop species and their closely related wild species strongly overlap with the Vavilov centers (Pironon et al., 2020). Identification of both crop and wild species diversity hotspots will provide opportunities for identifying and applying more focused conservation strategies for CWRs.

Considering CWRs have been neglected for years and there are many endangered species assessment of national and/or global *in-situ* resources to identify which CWRs are endangered or becoming extinct, whilst screening areas that are rich in wild crops and biodiversity (Hübner and Kantar, 2021) is crucial for protecting CWRs. For example, an assessment of wild banana species (*Musa* spp.) found that 11 out of 59 CWRs are vulnerable and another nine are endangered (Mertens et al., 2021). Khoury et al. (2019), found that of 600 CWR taxa assessed 7% may be critically endangered in their natural habitat and 50% may be endangered. These assessment programs involve a ‘gap analysis’ whereby the currently known and available CWR taxa (*in-situ/ex-situ* resources) are evaluated for their ability to provide future biodiversity to improve food security (Zair et al., 2021). By conducting a thorough gap analysis, Ng'uni et al., 2019., found that 459 CWR taxa out of a national Zambian inventory of 6305 taxa should now be included as part of their conservation and sustainability CWR checklist, with 59 to be specifically prioritised for future food security. The identified taxa represented an agriculturally significant group that was selected due to a shift in socio-economic values to ensure the nation’s food security in the oncoming years. Several nations have conducted their own gap analysis to ensure food security (Contreras-Toledo et al., 2019; Ng'uni et al., 2019; Tas et al., 2019; González-Orozco et al, 2021; Khaki Mponya et al., 2021; Rahman et al., 2021) and globally ten new *in-situ* conservation sites have been recommended as conservation zones to help achieve global food demand by expanding the *in-situ/ex-situ* resources (Zair et al., 2021).

To successfully establish *in-situ/ex-situ* resources to maintain and improve biodiversity, nations must create an inventory of all known plant taxa. These inventories provide a preliminary resource for the identification of critical taxa, such as CWRs (Teso et al., 2018; Allen et al., 2019; El Mokni et al., 2022). Whilst it is important for each nation to conduct an internal inventory, an unbiased global-scale inventory is also critical to establish CWR taxa. Vincent et al. (2013), originally created a global inventory of important CWR taxa, totaling 1667 taxa, divided between 37 families and 108 genera (Vincent et al., 2013). These inventories serve as the foundation for *in-situ/ex-situ* conservation, as they represent a ‘living’ CWR databank. However, as these taxa are truly wild, they will continue to evolve, and as such inventories only represent a snapshot of the population from the time of sampling, and recurring sampling is required to update inventories. A list of major global and national inventories is shown in **Table 2**.

**Table 2:** A list of major global and national CWR plant inventories.

Inventory name and details	Location assessed	Reference
Globally important CWR taxa	Global	(Vincent et al., 2013)
Important CWR taxa of Mexico	Mexico	(Contreras-Toledo et al, 2018)
National inventory of CWR in Spain	Spain	(Teso et al., 2018)
National inventories of CWR	Portugal	(Brehm et al., 2008)
CWR in USA	USA	(Khoury et al., 2013)
Enhancing and stating the UK CWR inventory	UK	(Fielder et al., 2015)
Prioritised CWR inventory of Italy	Italy	(Landucci et al., 2014)
Enhancing the CWR inventory of Scotland	Scotland	(Fielder et al., 2016)
Setting conservation priorities for CWR in the Fertile Crescent	Fertile Crescent	(Zair et al., 2018)
Prioritised inventory for Tunisia	Tunisia	(El Mokni et al., 2022)
CWR inventory of South, West and North Africa	South, West and North Africa	(Lala et al., 2018; Allen et al., 2019; Nduche et al., 2021)

## Platforms: Tools for accessing, managing or utilising CWR data and metadata

Several platforms have begun to emerge with the explicit purpose of user-friendliness, designed to aid breeders and scientists alike (Raubach et al., 2021) to facilitate accessibility to CWR resources, including germplasm and genomic data (**Table 1**). These platforms attempt to solve the most common challenges in handling high throughput data from phenotyping to genotyping: 1) data format, 2) data sharing, 3) data versioning, and 4) historical data (Raubach et al., 2021). For example, GRIN-global (https://www.grin-global.org/ ) is open-source software for genebank workers to create and manage a genebank’s data. Genesys and CGIAR are also examples of genebank platforms/databases (as discussed in the *ex-situ* section) that have been developed at a global scale to efficiently store and categorise data and facilitate the access and conservation of plant species including CWRs. Several other platforms are also available (discussed in the following sections) for visualizing, managing, accessing and storing large datasets related to crops and their relatives.

### Software/tool-based platforms

Software/tool-based platforms are essential for data visualisation or organisation and help to gain a better understanding of the accessions stored in genebanks. For example, the Crop wild phylorelative platform (CWP in **Table 1**) (Viruel et al., 2021) helps to predict the phylogenetic distance (through housekeeping genes or whole genome analysis) and cytogenetic compatibility for breeding programs to help estimate the CWR gene pool classification (Brozynska et al., 2016; Viruel et al., 2021). Alternatively, plaBiPD provides an online platform that visualizes the phylogenetic relationship of genome sequences of flowering plants including CWRs. Furthermore, the associated Mercator online tool allows for the assignment of functional annotations to land plant protein sequences (Schwacke et al., 2019; Bolger et al., 2021).

### Database management platforms

Database management tools provide a quick and easy to use platform for the access, management and use of data derived from breeding programs, research studies and trait identification programs using both CWRs and farmed crops. The genotyping platform Germinate v3 (**Table 1**) (Shaw et al., 2017; Raubach et al., 2021) provides a rapid directory for importing and exporting plant genetic data such as erm plasm, markers, traits and locations. Germinate v3 has showcased its usefulness in breeding efforts that involve CWRs, specifically those associated with the Crop Trust Crop Wild Relatives project (https://www.cwrdiversity.org ). Currently, Germinate v3 (20^th^ of April, 2022) contains the directories for CWR taxa: Cowpea (~13100 germplasms), Finger Millet (~1600 germplasms), Grass Pea (~5600 germplasms), Pigeonpea (~2900 germplasms), Chickpea (~23500 germplasm), Alfalfa (~2700 germplasms), Carrot (248 germplasms), Pearl Millet (~2400 germplasms), Barley (~33200 germplasms), Wheat, Sorghum (~2800 germplasms), Eggplant (~3300 germplasms), Rice (~4900 germplasms) and Sunflower (~7900 germplasms) and DIIVA (~2900 germplasms). The use of Germinate has been employed in recent CWR studies. For example, Kouassi et al., 2021., generated interspecies hybrids with eggplants and nine related CWRs. The successfully generated hybrid lines were genotypically and phenotypically screened, wherein it was established that the drought tolerance traits were controlled by genes that are in linkage disequilibrium or have pleiotropic effects. The phenotypic characteristics have been stored in Germinate to provide access to both the user and breeders (Kouassi et al., 2021). Furthermore, Germinate also provides evaluation data of breeding programs. Metwally et al., 2021., generated 13 new superior F_10_ lines of cowpea by crossing CWRs, improving seed yield and seed quality, as well as introducing earlier maturation. The two datasets which cover 11 different traits for 15 cowpea accessions (total of 2640 data points) were uploaded to Germinate for visualization or downloads (Metwally et al., 2021).

Breeding and research resources are widely available for several crop species such as GrainGenes for wheat, barley, rye and oat (Blake et al., 2019), MaizeGDB for maize (Portwood et al., 2019) and SoyBase for soybean (Grant et al., 2010). These databases primarily host and facilitate the exploration of detailed breeding, pedigree, QTL and molecular information across crop populations. Whilst genomic information regarding CWRs may be presented in these databases, particularly in the case of family-wide databases such as the Sol Genomics Network for Solanaceae (Fernandez-Pozo et al., 2015), they are deposited with no tools for comparative analysis. The development of integrated tools accessible in comprehensive databases is needed to facilitate direct comparisons between wild and domesticated individuals.

### Genomic databases

The PLAZA platform holds genomic data of both monocots and dicots. This platform compares the genomic data of submitted dicots and monocots to centralized genomic databases (Van Bel et al., 2022). The submitted genomic data is represented as an interactive phylogenetic tree style figure that links to a bioinformatic ‘workbench’. The workbench includes tools such as gene family plots, collinearity statistic tools, localization tools and direct BLAST tools to the PLAZA protein sequences. Similarly to PLAZA, CerealsDB is a specific database platform for cereals like wheat (Wilkinson et al., 2020), providing several key features such as a SNP database for Axiom^®^ 820K and 35K SNP arrays, KASP probes, iSelect Arrays, TaqMan^®^ probes. The database is curated to provide agronomically important SNPs (e.g. flowering time associated markers). Furthermore, database platforms such as the Brassica information portal (Brassicaceae) (Eckes et al., 2017) and the Genome database for Rosaceae (Rosaceae) (Evans et al., 2013) have been established as a way to collate and exchange open source information relating to the Brassica and Rosaceae genomes and genetics, respectively, although the databases do not contain CWR resources directly, many of the projects included do include CWR resources. The Legume Information System and Legume Federation project provides an excellent collection of genomic and variant data for over 15 crop species, with a large range of accompanying CWR data (Dash et al., 2016).

### Platform models that assist in data handling

A major issue in integrating informatics is a standardised model for data handling, especially as the information regarding the CWR conservation status and breeding programs is diverse and dispersed (Moore et al., 2008). These challenges can be identified by understanding the findable, accessible, interoperable and reusable (FAIR) curation and annotation of minor and underutilized crops (Andrés-Hernández et al., 2021). To address this, the European Crop Wild Diversity Assessment and Conservation Forum developed the Crop Wild Relative Information system (CWRIS) that incorporates an eXtensible Markup Language schema to aid data sharing and exchange. This system integrates with more partitions data into taxon-, site-, and population-specific elements, allowing for the integration with standard conservation biology (Kell et al., 2007; Kell et al., 2008; Moore et al., 2008). CWRIS was developed to provide access of the CWR data to a broader user community such as plant breeders, conservation and rehabilitation site managers, government, biologists and the wider public (Kell et al., 2007). CWRIS has since been integrated into GRIN-Global (https://npgsweb.ars-grin.gov/gringlobal/taxon/taxonomysearchcwr ), as the website is no longer being maintained or updated.

## Pangenomes to capture CWRs genetic variation

In recent years, advances in genome sequencing and bioinformatic tool development have extended the means to fully catalogue genetic variation among domestication and CWR populations through the construction of pangenomes (Bayer et al., 2020; Jayakodi et al., 2021; Tay Fernandez et al., 2022). Pangenomes achieve this by providing a comprehensive genomic reference to which both small variants, including single-nucleotide polymorphisms (SNPs), and structural variants, including presence/absence variation of large nucleotide sections (PAVs), can be identified across diverse populations (Danilevicz et al., 2020). In addition, analysis of pangenomics allows for the more accurate predication of underlying genetics that are associated with phenotypic variation, such as transposable elements, recombination and double-stranded break/repair (Saxena et al., 2014; Dolatabadian et al., 2020; Song et al., 2020). As pangenomes excel in capturing large structural variation, as is increasingly found between highly divergent populations, they are ideally suited for the comparison of domesticated genomes to CWR taxa to capture ‘wild genes’ that would be overlooked when using a traditional reference genome (Khan et al., 2020). For example, a pangenome assembly of *Brassica oleracea* with 87 domesticated accessions (Bayer et al., 2021b) identified 58,347 genes across all individuals in comparison to a study that included 8 domesticated accessions and 1 CWR (Golicz et al, 2016) (8 landraces and 1 CWR), which identified a higher number of genes (63,865) (Golicz et al., 2016; Bayer et al., 2021b). Similar findings have been shown in sorghum (Tao et al., 2021) and rice (Xu et al., 2012), where the inclusion of CWR individuals led to large increases in the breadth of genes uncovered.

Beyond capturing more genes, the addition of CWR to pangenomes facilities the identification of novel SNPs and PAVs that are not found in domesticated populations. For example, Mace et al., 2021 performed comparative analysis in sorghum to quantify the ‘contribution of CWR diversity’ by establishing the average total number of SNPs per genotype. They found that wild/weedy species contained about one SNP every 763 bp compared to landraces that contained one SNP every 1,282 bp and inbred lines containing one SNP every 1,543 bp (Mace et al., 2021). Lam et al., 2010 also performed a comparative study between 17 wild and 14 cultivated soybean genomes showed higher diversity of SNPs and PAVs among wild species in compared to cultivated. In total, they found 6,318,109 SNPs and 186,177 PAVs, with the CWR genomes carrying 34.66% more SNPs (Lam et al., 2010). This is a clear indication that through optimising our agriculturally important crops, their respective genetic diversity has been reduced and CWR make promises to widen selection diversity (Nelson et al., 2018; Bailey-Serres et al., 2019).

## Machine learning and CWRs

The application of machine learning (ML) has proven its efficiency in handling huge amounts of data and is becoming more popular in various plant science fields including gene identification and classification, and biodiversity analysis (Bayer et al., 2021a). For example, in Arabidopsis a ML model was developed to identify candidate stress-related genes by comparing whole genome expression data between the control and stress samples (Wegrzyn et al., 2014). In soybean, a ML model was developed to predict agronomically important traits, including yield, protein, oil, moisture and height, using SNP markers (Liu et al., 2019). Similarly, Ma et al., 2018., successfully developed a ML model to predict eight phenotypic traits among 2000 wheat individuals using 33,709 DArT (Diversity Array Technology) markers (Ma et al., 2018). ML is now also being used to predict mature yield in early development using a combination of image and genotype data (Danilevicz et al., 2021; Danilevicz et al., 2022). Recently ML models were developed for identification of core and dispensable genes in *Oryza sativa* L. and *Brachypodium distachyon* (L.) P. Beauv. using existing pangenomic information. The significant potential of these models is to identify core and dispensable genes in a new species without construction of pangenome (Yocca and Edger, 2022), such approaches can facilitate and speed up genes identification in new cultivated and wild species.

Understanding and usage of environmental conditions, in particular of CWR populations helps in selecting individual populations for the specific introgression goal. CWRs and landraces have occupied local niches (e.g., hot vs. cold regions) and have been shaped by natural selection (Cortés and López-Hernández, 2021), and these traits can be easily tracked when considering collection environmental site parameters. For example, Ariani et al, 2018, by using ∼20,000 SNPs across 249 accession of wild *Phaseolus vulgaris*, identified 5 geographically distinct subpopulation, which mostly affected by temperature and rainfall of the regions (Ariani et al., 2018) Berny Mier Y. Teran et al., 2020, also documented that the lines driven from wild parents from the lower rainfall regions produced higher yield in both drought and watered conditions in compare to lines driven from domesticated parents (Berny Mier Y. Teran et al., 2020). Using ML algorithms is also a powerful approach to combine information of germplasm resources and environmental conditions for identification of candidate germplasms with traits of interest. This approach, finding adaptative traits based on environmental parameters, is known as FIGS (Focused Identification of Germplasm Strategy) (Khazaei et al., 2013). Several ML models based on the FIGS approach have been successfully developed and used for identifying germplasm of interest (**Table 3**). For instance, the identification and classification of *Vicia faba* genetic resources with traits related to drought tolerance (Khazaei et al., 2013). Similarly, in wheat, ML algorithms used for analysing accumulative stem rust trait data (1988-1994), and geographical data of accessions (including landraces and improved accessions) screened for stem rust over 2,000 collection sites revealed an association between the geographic distribution of resistance accessions and environmental variables at collection sites (Bari et al., 2012). Another ML model was successfully developed to predict stripe rust resistance in wheat, based on the stripe rust scores of 725 wheat landrace accessions with collection site information associated with 2,910 accessions in the ICARDA genebank (Bari et al., 2014). Genetic diversity analysis among 80,000 wheat accessions (including 3,903 wild relatives) also revealed landraces with unexplored diversity and genetic footprints defined by regions under selection (Sansaloni et al., 2020). ML has facilitated the study and discovery of several genetic resources with agronomically valuable traits in crops. There are also “global database for the distribution of wild relatives” (https://www.gbif.org/dataset/07044577-bd82-4089-9f3a-f4a9d2170b2e ) which includes the distribution data of crop wild relatives that can be used to extract geographical information and potential environmental conditions for CWRs.

**Table 3:** Case studies of the most recent applications of CWRs for crop improvement.

CWR	Application	Outcome	Reference
CWR of Cinnamomum, Piper, Vigna and Oryza in Sri Lanka	ML models to simulate the potential distribution across nine CWR species	The model was able to identify highly vulnerable species to climate change and predict the potential decrease in their suitable habitat by 2050. The study also identifies potential CWR rich areas for future *in-situ* conservation.	(Ratnayake et al., 2021)
ICARDA genebank barley accessions	FIGS *via* ML models	Providing predictive characterization for entire ICARDA barely collection	(Azough et al., 2019)
Wild blueberry	ML algorithms for yield prediction by evaluating bee species composition and weather factors	Prediction (with 93% accuracy) showed bee species composition and weather are significant in yield variability while wet rainy springs will greatly reduce blueberry yield.	(Obsie et al., 2020)
Wild cacao	Using ML model for surveying canopy and vegetation assessments	92% of classification accuracy for the structural attributes of the canopy	(Duarte-Carvajalino et al., 2021)
Large collection of *Vicia faba* L.	ML models used to evaluate FICS approach for identification of traits related to drought	The model was successful to indicate leaflet, canopy temperature and relative water content are important traits for drought-tolerance selection.	(Khazaei et al., 2013)
*Solanum pimpinellifolium*	Genome editing (*de novo* domestication	Produced a modified version of the wild *S. pimpinellifolium* which displayed a 10 times increase in the number of fruit and a 3 times increase in fruit size. The fruit also contained 500% more lycopene compared to the commonly cultivated *S. lucopersicum*.	(Ariani et al, 2018)
*Physalis pruinose*	Genome editing (*de novo* domestication	Edited orthologues of cultivated tomato in the distant relative *P. pruinose* to improve plant architecture, flower production and fruit size.	(Lemmon et al., 2018)
*Oryza alta*	Genome editing (*de novo* domestication	Established the first ever polyploid rice by genome editing the allotetraploid relative *O. alta*.	(Yu et al., 2021)
*Aegilops tauschii*	Association genetics with resistance gene enrichment sequencing (AgRenSeq)	Developed the AgRenSeq methodology and identified two novel wheat stem rust resistance genes, *Sr46* and *SrTA1662*, in a wild wheat progenitor.	(Arora et al., 2019)
*Solanum americanum*	Resistance gene enrichment sequencing and single-molecule real-time sequencing (SMRT RenSeq)	Identified the genome-wide repertoire of nucleotide-binding leucine-rich repeat type R genes in the wild *S. americanum* and cloned *Rpi-amr3i*, a novel R gene for potato late blight.	(Witek et al., 2016)
*Oryza rufipogon*	Genome editing	Optimised an efficient transformation system in wild rice, aiding future genome editing efforts including *de novo* domestication.	(Xiang et al., 2022)
*Solanum peruvianum*	Genome editing	Developed a genome editing approach using protoplast regeneration for the tetraploid wild tomato relative.	(Lin et al., 2022)

## Limitation to uses of CWRs within breeding programs

There are many challenges that still prevent the wide-spread use of CWRs as a source of superior alleles that can be incorporated into elite cultivated germplasm. The relatedness, compatibility and crossability of CWRs to their cultivated counterparts is one issue largely inhibiting the straightforward introduction of CWR traits through traditional breeding. For example, in cotton highly disease resistant sources were identified in wild diploid species, including *Gossypium. longicalyx* J.B. Hutch. & B.J.S. Lee; *G. somalense* (Gürke) J.B. Hutch.; *G. stocksii* Mast.; *G. arboreum* L.; and tetraploid species of *G. barbadense* L. (Yik and Birchfield, 1984); however due to genetic incompatibility, ploidy, climbing growth habit, photoperiodism, and agronomic issues breeders were unable to use these resources. Later, through the development of three-species hybrids, researchers were successfully able to introduce donor plants which were fertile and had reniform nematode resistance (Robinson et al., 2004; Konan et al., 2007).

Furthermore, trait identification and selection might be challenging and significantly affected by environment as there are radically different selection regimes in a wild state/region compared to a domesticated state/region while a trait can be useful in a domesticated state (and selected for) may not be useful in the wild and vice-versa. For example, Parker et al. (2020), suggested the decreased-pod dehiscence (PD) trait among domesticated haplotypes of common bean is as a result of the different fitness landscape imposed by domestication, where stronger selection pressure were used against PD in arid condition of North Mexico compared to tropical lowlands (Andes), where environmental humidity masks susceptibility to PD and reducing selection pressure against it (Parker et al., 2020). It is also often challenging to accurately evaluate the yield of CWRs since they can display growth forms or traits that are difficult to manage, for example the wild progenitor of common bean has naturally dehiscent seed pods, making yield measurements arduous to obtain, and has a larger, less compact growth habit that is far less suitable for cultivated environments compared to cultivated common bean (Koinange et al., 1996). Even if beneficial wild derived traits are introgressed into elite material, they can often have a negative effect on yield or yield-related traits, through linkage drag. A common example is the introduction of biotic stress tolerance genes, for example disease resistance genes, which improve some resistance/tolerance but are detrimental to other agronomic traits (Brouwer and St Clair, 2004; Summers and Brown, 2013) Furthermore, after introducing genetic material from CWRs into an elite background, problems with sterility, often seen at the F_1_ or BC_1_ generation, can arise (Wang et al., 2020; Bohra et al., 2022).

There are also a number of challenges of CWR application in breeding that have been eased by availability of more genomic resources, and advances in laboratory techniques, as discussed in the following section. These include lack of information of gene-trait relationships in wild species, uncertainty of how allelic combinations will be expressed in different cultivated crop backgrounds and difficulties of transferring genes of interest into crops (Dempewolf et al., 2017).

## Modern breeding and CWRs

There are now avenues to harness CWRs and overcome some of these barriers. For instance, wild-derived genes conferring desirable alleles can now be introduced through precise genome editing into elite backgrounds without the need for lengthy introgression regimes, bypassing the barriers of linkage drag and reduced fertility that so often complicate the use of CWRs (Bohra et al., 2021). These modern approaches, utilising the advances in genomics and genome editing, provide promising pathways to overcome long-standing challenges and push CWRs to the forefront of crop improvement. **Table 3**, included examples of successful application of CWRs for crop improvement *via* modern breeding approaches.

Genomics provides an avenue to explore the genetic diversity in CWRs and identify agronomically valuable genes or QTL. Sequencing CWRs followed by *de novo* assembly can generate reference assemblies that underpin downstream applications, such as the functional characterization of genes and targeted genome editing. Although initially lagging behind cultivated crop genomes, a number of CWRs assemblies are now becoming available, including relatives of barley, rice, soybean, tomato and wheat (Brozynska et al., 2016; Bohra et al., 2022). Often in combination with high-throughput phenotyping, these genome assemblies have enabled the identification of several important genes and QTL from CWRs, for example numerous disease resistance genes in wheat (Yahiaoui et al., 2009; Periyannan et al., 2013; Saintenac et al., 2013) and QTL associated with oil content in soybean (Zhou et al., 2015). High-quality assemblies based on third generation long read sequencing are now becoming the standard for reference genomes in major crops. Advances in long-read sequencing in terms of increased accessibility and lower price points, will be vital for the construction of high-quality long read assemblies in a broad range of CWRs, which will unlock an arsenal of beneficial CWR genetic diversity ready to be harnessed for crop improvement.

There are also recent genomic methodologies that have been developed to identify genes linked to specific traits; for instance resistance gene enrichment sequencing (RenSeq) is a methodology that targets, enriches and sequences *R* genes within any plant genome based on common *R* gene motifs (Jupe et al., 2013). To date, it has been used to capture nucleotide-binding-site leucine-rich repeat proteins (NLRs), receptor-like proteins (RLPs) and receptor-like kinases (RLKs), which represent the largest families of *R* genes (Jupe et al., 2013; Lin et al., 2020). Since its initial development, RenSeq has been combined with other approaches, including ethyl methanesulfonate (EMS) mutagenesis (MutRenSeq), single-molecule real-time sequencing (SMRT RenSeq) and association genetics (AgRenSeq). These combined workflows have rapidly identified and cloned causative *R* genes in a wild potato relative (Witek et al., 2016), wheat (Steuernagel et al., 2016), wild diploid wheat (Arora et al., 2019) and rye (Vendelbo et al., 2022). RenSeq is a promising alternative to whole genome sequencing for large scale *R* gene identification, and if utilised in CWRs, has the potential to rapidly expand the *R* gene arsenal used for breeding disease resistant cultivars. Notably, AgRenSeq does not rely on a reference genome (Arora et al., 2019), therefore it is extremely applicable to CWRs that are yet to have a reference assembly, but whose cultivated counterpart has well characterised *R* genes.

While there has been rapid progress within the field of plant genome editing, the application within CWRs has been far slower. The limited genomic resources for many CWRs serves as an initial barrier, then the lack of functionally characterized gene targets and easy delivery system for those targets proves arduous. In spite of these challenges, one innovative application of CRISPR recently proposed is the manipulation of genes controlling important agronomic traits, for example plant architecture genes, while purposefully retaining valuable wild-derived traits such as stress tolerance or improved nutritional quality; in essence, the domestication of a CWR or landrace that has never been cultivated. This approach, termed *de novo* domestication, can produce new crops from a CWR in a matter of generations through genome editing technology (Gasparini et al., 2021). Using a wild tomato relative, Zsögön et al., 2018., edited four key tomato domestication genes, *SELF*-*PRUNING*, *OVATE*, *FRUIT WEIGHT 2.2* and *LYCOPENE BETACYCLASE*, to produce an engineered tomato crop boasting increased fruit number and size compared to the wild parent, and vastly improved nutritional quality compared to cultivated tomato (Zsögön et al., 2018). A similar approach was undertaken in the orphan crop groundcherry, a distant tomato relative, whereby productivity traits including plant architecture, flower production and fruit size were improved by editing known tomato orthologues with CRISPR-Cas9 (Lemmon et al., 2018). One ambitious study utilised *de novo* domestication to develop the first ever polyploid rice crop, through the rapid domestication of an allotetraploid wild rice, *Oryza alta* (Yu et al., 2021). This has demonstrated a feasible route to create polyploid versions of diploid crops, which are said to benefit from genome buffering *via* gene redundancy, hybrid vigour and environmental fortitude (Mason and Batley, 2015). As researchers characterise more genes related to key domestication traits in model or major crops and high-quality CWR genome assemblies are generated, the potential for editing these genes in CWRs skyrockets, leading to the possible creation of new crops through *de novo* domestication. Furthermore, simultaneously identifying and cataloguing agronomically beneficial traits in CWRs will greatly enhance our ability to exploit wild genetic diversity, meaning *de novo* domesticated crops will be more nutritious and climate resilient than their cultivated relatives.

Despite the promising potential of *de novo* domestication, one of the major challenges preventing the widespread deployment of CRISPR in CWRs, and therefore *de novo* domestication, is the delivery system of the genome editing reagents. Even for elite cultivars, quick and easy methods for delivery that are widely transferable between species remain elusive (Zhan et al., 2021). The most popular DNA delivery approaches include agrobacterium-mediated delivery, which utilises the soil pathogen *Agrobacterium tumefaciens* to transfer DNA into the host genome, and biolistic or micro-projectile-mediated delivery, where the donor DNA is mechanically forced into the host cells (Ran et al., 2017). However, these methods come with certain limitations. *Agrobacterium*-mediated delivery is hindered by its inability to introduce small donor fragments, its difficulty in preventing plasmid integration and thereby producing a transgenic plant, and is dependent on the genotype of the recipient, particularly for monocot plants (Ran et al., 2017). While biolistic methods provide some advantages over *Agrobacterium*-mediated delivery, for example the delivery of multiple targets, its use is lower than expected due to issues with multiple copies of the transgene being incorporated into the host, resulting in altered gene expression or complete silencing. Efficient delivery methods using these approaches, after significant optimisation, have been established in model plants and select major crops. However, such methods are not easily transferrable to CWRs, as they often represent a diverse set of morphotypes which introduces unique challenges hindering delivery. On top of this, CWRs are also difficult to regenerate, further complicating the transformation process (Zhu et al., 2020).

Several alternative approaches for reagent delivery which were initially developed in animal cells, are being explored in plants (Ghogare et al., 2021). For example, a biolistics approach using nanoparticles offers a less harmful delivery method compared to larger microparticles, which may reduce delivery damage, a common issue encountered in plants due to the presence of a cell wall (Zhang et al., 2019; Cunningham et al., 2020). Most excitingly, delivery mediated by viral vectors can completely bypass the need for regeneration which is an extremely promising prospect for editing hard to regenerate CWRs, however this method is limited by its delivery capacity (Shan-e-Ali Zaidi and Mansoor, 2017). Novel delivery methods will help to overcome the barriers preventing widespread plant transformation and reduce the amount of optimisation needed. In doing so, efficient genome editing in CWRs will be one step closer.

Another potential approach for CWRs utilization in breeding schemes is through speed breeding. The concept of speed breeding revolves around manipulating the photoperiod (e.g. 12 hr extended to 22 hr) and temperature in a controlled growth facility to rapidly produce multiple crop generations per year (Watson et al., 2018). Through speed breeding, the genetic background of cultivars can be fixed in an accelerated timeframe, a process which usually takes years of inbreeding. Speed breeding has been tested and effectively produced multiple generations in a single year for crops such as barley, canola, chickpea, pea, rice, sorghum and wheat (Espósito et al., 2012; Rizal et al., 2014; Watson et al., 2018; Nagatoshi and Fujita, 2019; Rana et al., 2019). In the absence of precise genome editing, desirable traits from CWRs which are introgressed into elite cultivars through traditional breeding will often bring with them unwanted deleterious alleles. Hence, speed breeding can facilitate the quick growth of multiple generations, allowing undesirable traits to be selected against, and for these new varieties to reach a stable genetic background. In addition, speed breeding would benefit alternative approaches to domesticate CWRs without the use of CRISPR, such as germplasm conversion (Stephens et al., 1967; Rosenow et al., 1997; Klein et al., 2016). Germplasm conversion involves the alteration of germplasm through crossing, multiple rounds of selection for various traits and inbreeding to become well-adapted to new environments while also having favourable agronomic traits (Stephens et al., 1967). Extensive germplasm conversion has been done in Sorghum to transform numerous exotic varieties into early-maturing and dwarf-height varieties that are adapted for cultivation in the US or other temperate regions (Stephens et al., 1967; Rosenow et al., 1997; Klein et al., 2016). As an alternative to genome editing, germplasm conversion could be harnessed to introduce important agronomic traits into CWRs through hybridization and then followed by marker-assisted selection (MAS). The advantage of this over genome editing is that specific knowledge of the target sequences is not required, only knowledge of the genomic region conferring the domestication trait/s. However, it is likely that this method would be more laborious and time consuming compared to genome editing approaches, as several generations are usually required to achieve the final product. Therefore, exposing these CWRs to speed breeding conditions may help to mitigate the time required for cycling multiple generations that is necessary for effective germplasm conversion of CWRs into commercially viable crops (Bhatta et al., 2021).

## Conclusion

Crop wild relatives have remained under-utilised during crop domestication and intense crop breeding, despite the fact they harbour beneficial traits such as disease and pest resistance, and tolerance to abiotic stresses. CWRs have the potential to widen selection sources for breeders beyond the existing variation among cultivated crops to meet future foods’ quality and quantity demands. A multi-resource integrative approach that utilises many of the resources outlined here will enable CWRs to be effectively used as a source of valuable genetic diversity. For example, ML strategies based on FIGS in combination with genomic and pangenomic resources that capture the gene diversity that exists in CWRs, will help to rapidly identify adaptative traits based on environmental parameters which will in turn guide the identification of genes underpinning these traits. However, realisation and utilisation of the full potential of the genes and diversity presented in CWRs will ultimately depend on the availability of resources and experimental techniques to support breeding programs (Hajjar and Hodgkin, 2007). There are a number of resources and databases that both researchers and breeders can benefit from, but ongoing efforts are crucial to keep these data well organised and up-to-date. This is only possible with the great collaboration between ecological/biological conservation sectors, who manage CWR ex/in -situ conservation and prevent extinction, researchers in the field of computer science, plant biology, for example plant genomics and agricultural industries, who assist with identification of traits/genes of interest among CWRs and only with this multidisciplinary effort is there a chance to guarantee the future food demands.

## Author contributions

ST and JB conceptualized the review. ST wrote the main text with additions from WT, JZ, JM, DE and JB. DE and JB edited the paper. All authors contributed to the article and approved the submitted version.

## Funding

This work was funded by the Australian Research Council projects DP200100762, DP210100296 and the Grains Research and Development Corporation (UWA1905-006RTX).

## Acknowledgments

WT would like to acknowledge the support of the Grains Research and Development Corporation.

## Conflict of interest

The authors declare that the research was conducted in the absence of any commercial or financial relationships that could be construed as a potential conflict of interest.

## Publisher’s note

All claims expressed in this article are solely those of the authors and do not necessarily represent those of their affiliated organizations, or those of the publisher, the editors and the reviewers. Any product that may be evaluated in this article, or claim that may be made by its manufacturer, is not guaranteed or endorsed by the publisher.
